# Gastric microbiota in gastric cancer: Different roles of *Helicobacter pylori* and other microbes

**DOI:** 10.3389/fcimb.2022.1105811

**Published:** 2023-01-10

**Authors:** Yang Guo, Xue-Shan Cao, Meng-Ge Zhou, Bo Yu

**Affiliations:** ^1^ Department of Dermatology, Institute of Dermatology, Peking University Shenzhen Hospital, Shenzhen Peking University-The Hong Kong University of Science and Technology Medical Center, Shenzhen, China; ^2^ College of Life Science and Oceanography, Shenzhen University, Shenzhen, China; ^3^ Department of Epidemiology and Biostatistics, Institute of Basic Medical Sciences Chinese Academy of Medical Sciences, School of Basic Medicine Peking Union Medical College, Beijing, China

**Keywords:** gastric cancer, gastric microbiota, *Helicobacter pylori*, carcinogenesis, dysbiosis

## Abstract

Gastric cancer (GC) is one of the leading causes of cancer-related deaths worldwide. The gastric microbiota plays a critical role in the development of GC. First, *Helicobacter pylori* (*H. pylori*) infection is considered a major risk factor for GC. However, recent studies based on microbiota sequencing technology have found that non-*H. pylori* microbes also exert effects on gastric carcinogenesis. Following the infection of *H. pylori*, gastric microbiota dysbiosis could be observed; the stomach is dominated by *H. pylori* and the abundances of non-*H. pylori* microbes reduce substantially. Additionally, decreased microbial diversity, alterations in the microbial community structure, negative interactions between *H. pylori* and other microbes, etc. occur, as well. With the progression of gastric lesions, the number of *H. pylori* decreases and the number of non-*H. pylori* microbes increases correspondingly. Notably, *H. pylori* and non-*H. pylori* microbes show different roles in different stages of gastric carcinogenesis. In the present mini-review, we provide an overview of the recent findings regarding the role of the gastric microbiota, including the *H. pylori* and non-*H. pylori* microbes, in the development of GC.

## Introduction

Gastric cancer (GC) is one of the leading causes of cancer-related deaths worldwide, ranking fifth in incidence and third in mortality of cancers (Bray et al., 2018). According to World Health Organization International Agency for Research on Cancer (WHO-IARC), the annual burden of GC will increase to approximately 1.8 million new cases and 1.3 million deaths by 2040. Compared with those in 2020, the numbers of new cases and deaths will increase by approximately 63% and 66%, respectively (Morgan et al., 2022). *Helicobacter pylori* (*H. pylori*) infection is a critical risk factor for GC (Amieva and Peek, 2016) and *H. pylori* was classified by the WHO-IARC as a type I carcinogen (WHO-IARC, 1994). In recent years, sequencing-based studies focusing on microbiota have shown that patients with GC have gastric microbiota dysbiosis, including reduced microbial diversity, altered microbial community structure, altered compositions, and abnormal bacterial interactions (Gantuya et al., 2020; Kadeerhan et al., 2021). Furthermore, non-*H. pylori* microbes might also promote gastric lesions and even GC (Coker et al., 2017; Yu et al., 2017; Ferreira et al., 2018; Kadeerhan et al., 2021). The interactions between *H. pylori* and other microbes may be also involved in gastric carcinogenesis.

In the present mini-review, we aim to discuss the recent findings regarding the role of gastric microbiota, including *H. pylori* and non-*H. pylori* microbes, in the development of GC.

## *H. pylori* infection, eradication, and GC

*H. pylori* is a gram-negative, flagellated, microaerophilic bacterium belonging to the *Campylobacterota* phylum, which was first identified in 1982 (Warren and Marshall, 1983). *H. pylori* colonizes in the stomach and becomes the predominant microbe in stomach after infection (Schulz et al., 2018). In terms of the global epidemiology of *H. pylori* infection, according to a global meta-analysis (Hooi et al., 2017), there were about 4.4 billion *H. pylori*-positive cases worldwide in 2015. The prevalence rate of *H. pylori* infection varied by region, with the highest prevalence rate in Africa (70.1%, 95% CI: 62.6-77.7%) and the lowest prevalence rate in Oceania (24.4%, 95% CI: 18.5-30.4%). Furthermore, for the temporal trend of *H. pylori* infection, the prevalence in different regions is stable or decreasing, especially in the developed world and in children (Burucoa and Axon, 2017; Hooi et al., 2017).

*H. pylori* infection is considered a major risk factor for gastric carcinogenesis. Overall, a large-scale pooled analysis of case-control studies nested within prospective cohorts showed that *H. pylori* infection was associated with nearly six-fold increased risk of non-cardia cancer (Helicobacter and Cancer Collaborative Group, 2001). The mechanism that *H. pylori* induces GC has been explored (Ishaq and Nunn, 2015; Talebi Bezmin Abadi, 2016). First, *H. pylori* primarily triggers the transition from normal mucosa to non-atrophic gastritis and then initiates precancerous lesions (Díaz et al., 2018). The responses after infection are mainly mediated through the action of bacterial virulence factors, including cytotoxin-associated gene A (CagA), vacuolating cytotoxin A (VacA), and other outer membrane proteins (Díaz et al., 2018; Alipour, 2021). CagA has multiple effects on epithelial cells, including stimulating cell proliferation, reducing epithelial cell apoptosis, etc. (Saadat et al., 2007; Nagy et al., 2009; Buti et al., 2011). Additionally, inflammatory cells can be recruited and oxygen species-induced damage can be induced after CagA and the type IV secretion system (T4SS) activate the inflammatory signaling (Viala et al., 2004; Chaturvedi et al., 2011). VacA can also cause alterations of cells, such as vacuolization and promoting immune regulation (Willhite et al., 2003; Yang et al., 2022). Further, the urease production by *H. pylori* and the glandular atrophy induced by *H. pylori* infection lead to reduced acid production and shifts in gastric pH value. As a result, the bacterial colonization environment in the stomach changes and gastric microbiota dysbiosis may occur (Schulz et al., 2015; Noto and Peek, 2017). The above-mentioned effects promote GC development.

For *H. pylori*-positive cases, eradication therapy could be given (Fallone et al., 2016; Malfertheiner et al., 2017; Liu et al., 2018). The effect of *H. pylori* eradication therapy on the GC risk has been evaluated. You et al. reported that, based on a randomized trial with a follow-up of 7.3 years, *H. pylori* treatment resulted in statistically significant decreases in the combined prevalence of severe chronic atrophic gastritis, intestinal metaplasia, dysplasia, or GC (OR = 0.77, 95% CI: 0.62-0.95) (You et al., 2006). With a follow-up of 22 years for this randomized trial, this team found that the protective effect of *H pylori* treatment on GC incidence (OR= 0.48, 95% CI: 0.32-0.71) and GC death (HR= 0.62, 95% CI: 0.39-0.99) persisted 22 years post-intervention (Li et al., 2019). Additionally, a recent well-designed meta-analysis enrolling randomized controlled trials (RCTs) with 10 or more years of follow-up found that the GC incidence decreased significantly with *H. pylori* eradication therapy (RR=0.54, 95% CI: 0.41-0.72); on the other hand, eradication of *H. pylori* showed significant reductions in GC mortality (RR=0.66, 95% CI: 0.46-0.95) (Ford et al., 2022).

## *H. pylori* associated gastric microbiota dysbiosis

The gastrointestinal microbiota refers to microorganisms lived in the gastrointestinal tracts, which is critical to many aspects of human health (Clemente et al., 2012; Valdes et al., 2018). For human immune, the microbiota is key to the induction, training, and function of the host immune system (Belkaid and Hand, 2014; Ling et al., 2022). Regarding the gastric microbiota, due to the high acidity of the stomach, the human stomach was once assumed to be a sterile organ (Espinoza et al., 2018). However, *H. pylori* is able to colonize the human gastric mucosa and survive in the highly acidic environment of the stomach (Schulz et al., 2015). With the advent of novel techniques for analyzing the microbial community, the unique features of the gastric microbiota have been identified that the major microbes in the healthy human stomach environment are *Firmicutes*, *Bacteroidetes*, *Actinobacteria*, *Fusobacteria*, and *Proteobacteria* (Guo et al., 2020; Guo et al., 2021).

For *H. pylori*-infected individuals, the stomach is dominated by *H. pylori* and accordingly, the abundances of non-*H. pylori* microbes reduce substantially (Brawner et al., 2017; Das et al., 2017). In addition to the changes of microbial composition, other phenomena of gastric microbiota dysbiosis have also been found. For the microbial alpha diversity, Gantuya et al. reported that individuals infected with *H. pylori* showed significant decreased microbial diversity compared with *H. pylori*-negative individuals (Gantuya et al., 2019). Another study found that there was a negative association between the gastric microbiome diversity and *Helicobacter* abundance (Das et al., 2017). In addition to microbial alpha diversity, infection with *H. pylori* results in alterations of the microbial community structure (beta diversity). According to a population-based study, the *H. pylori* positive group and negative group were clearly separated according to beta diversity (Llorca et al., 2017). Furthermore, studies focusing on the microbial ecological interactions found shifts of the interactions between *H. pylori* and other microbes in the stomach environment. In detail, according to an Indian study using16S rRNA gene sequencing, the network analyses showed that *Helicobacter* had negative interactions with other microbes of the gastric microbiome (Das et al., 2017); another Chinese study reported similar findings (Guo et al., 2020). Regarding the numbers of interactions, Coker et al. found that *H. pylori* infection reduces the number of gastric microbiome interactions (Coker et al., 2017). However, all the above-mentioned findings were based on statistical analyses of sequencing data. Thus, we need more clinical data supporting current presented concept (Rivas-Ortiz et al., 2017).

For *H. pylori*-positive individuals, the *H. pylori* eradication could reverse gastric microbiota dysbiosis and exert beneficial effects on the gastric microbiota (Guo et al., 2022). Firstly, for the reduced gastric microbial diversity among *H. pylori*-positive cases, the diversity could increase significantly after successful eradication of *H. pylori* (Guo et al., 2020; Mao et al., 2021). Also, significant differences were observed for the microbial community structure (the beta diversity) following eradication (Guo et al., 2020; Sung et al., 2020b; Mao et al., 2021; Watanabe et al., 2021; Yuan et al., 2021). For the gastric microbiota composition, after removing *H. pylori* in the stomach environment, the gastric commonly dominant commensals are enriched (Guo et al., 2020; Shin et al., 2020). Different changes of specific microbes were reported, which may be resulted from different population, sequence methods, and sampling details. The common reported commensals included *Firmicutes*, *Streptococcus, Prevotella.*, etc. (He et al., 2019; Guo et al., 2020; Mao et al., 2021; Watanabe et al., 2021; Yuan et al., 2021). In terms of interactions between gastric commensal bacteria, a reduction in these interactions was reported after eradication of *H. pylori* (Sung et al., 2020b; Yuan et al., 2021), which were also based on statistical analyses of sequencing data and required further validation. Moreover, due to the development of bioinformatics, microbiota function could be predicted and analyzed. According to the bioinformatic analysis of functional capacity, the bacteria reproduction-related pathways are down-regulated and pathways of gastric acid secretion, etc. are up-regulated (He et al., 2019; Guo et al., 2020), indicating beneficial effect of eradication on the recovery of gastric microbiota. In combination with the prevention effect of *H. pylori* eradication on GC, the alterations in gastric microbiota after eradication may contribute to the reduction in GC risk; further studies with long-term follow-up are needed (Guo et al., 2022).

## The overall features of the gastric microbiota associated with GC

In recent years, the characterization of the gastric microbiota associated with GC has been identified, indicating that gastric microbiota dysbiosis occur in gastric carcinogenesis (Yang et al., 2021). In the year of 2009, the team of Prof. Engstrand compared the gastric microbiota of patients with GC and controls using the terminal restriction fragment length polymorphism (T-RFLP) and 16S rRNA gene cloning and sequencing. They found that diversity indices of GC microbiota were not significantly different from that in controls according to the T-RFLP. In terms of gastric microbiota composition of GC, the abundance of *H. pylori* was low and the GC microbiota was dominated by the following genera: *Streptococcus*, *Lactobacillus*, *Veillonella* and *Prevotella* (Dicksved et al., 2009). However, the sample size of this study was small (only ten patients and five controls); additionally, 16S rRNA sequencing technology and related procedures are not yet developed and extensively used, therefore this work is an initial investigation of this field.

In following decade, other findings have been reported. Firstly, the gastric microbial diversity alteration in GC has been the most focused topic. Several studies reported that compared with the gastritis status, gastric microbial diversity is significantly reduced; analyses showed that the microbial community structure (beta diversity) is significantly altered in GC patients (Coker et al., 2017; Ferreira et al., 2018). Similarly, according to studies based on comparison between GC tissues and non-cancerous tissues, GC tissues also have reduced diversity and shifted microbiota structure (Chen et al., 2019). However, the conclusions are inconsistent across studies. For instants, two studies showed that the alpha diversity of GC gastric microbiota was increased (Eun et al., 2014; Linz et al., 2017). The difference of results may be caused by different populations, sampling sites and stage of gastric disease.

In addition to microbial diversity analysis, with the development of bioinformatics, more in-depth analysis methods have been developed and used. The function prediction analyses have been applied to explore potential mechanisms of gastric carcinogenesis. The most studies did function prediction analyses using PICRUSt (Langille et al., 2013). Ferreira et al. identified the presence of a nitrosating microbial community in GC cases, indicating that nitrate-reducing bacteria may contribute to gastric carcinogenesis (Ferreira et al., 2018). Meanwhile, a switch towards purine metabolism, D-alanine metabolism, drug metabolism, etc. in GC were reported in another study (Coker et al., 2017). These findings suggested that the microorganisms in the stomach may contribute to the development of GC through specific functional effects. Similarly, these findings need further validation of mechanisms.

## The non-*H. pylori* microbes associated with GC

In addition to *H. pylori*, more and more studies have been focusing on other non-*H. pylori* gastric microorganisms. Similar to the bacterial driver-passenger model in the development of colorectal cancer (Tjalsma et al., 2012), the hypothesis of GC has been proposed that: *H. pylori*, as the “driver”, causes pathological changes of gastric mucosa and dysbiosis of gastric microbiota; with the progression of gastric lesions, the number of *H. pylori* decreases and the number of other microorganisms in the stomach, i.e. non-*H. pylori* microbes as the “passengers”, increases correspondingly. These non-*H. pylori* microbes play an important role in the pathogenesis of GC.

The above hypothesis has been confirmed in animal research. An animal study using hypergastrinemic insulin-gastrin (INS-GAS) transgenic mice found that compared with the specific pathogen free (SPF) INS-GAS mice, the duration of gastric lesions development was longer for germ-free INS-GAS mice; compared with INS-GAS mice infected with *H. pylori* only, INS-GAS mice with complex gastric microbiota had more severe gastric lesions and an earlier onset of gastrointestinal intraepithelial neoplasia (Lofgren et al., 2011). Another INS-GAS mice-based study reported that INS-GAS mice coinfected with *H. pylori* and other intestinal bacteria had a higher rate of development of gastrointestinal intraepithelial neoplasia than those infected with *H. pylori* alone (Lertpiriyapong et al., 2014). These findings indicate the potential role of non-*H. pylori* microbes and the interactions between *H. pylori* and non-*H. pylori* microbes in gastric carcinogenesis.

More researchers are paying attention to human studies as the hypothesis is supported in animal studies. In a population-based study using the 16S rRNA gene sequencing method, compared with individuals with gastritis, GC showed gastric microbiota dysbiosis and a lower abundance of *Helicobacter* and the over-representation of intestinal commensals was seen in GC gastric microbiota. In detail, 16 enriched taxa and 13 depleted taxa in GC according to the LEfSe analysis (Ferreira et al., 2018). Another study comparing gastric microbiota of GC patients and superficial gastritis reported that 21 bacterial taxa were enriched in GC and 10 bacterial taxa were depleted in GC. Specifically, enrichment of oral microbes was observed in the stomach of GC (Coker et al., 2017). In addition to above two cross-sectional studies, a cohort study with a 4-year follow-up reported that *Helicobacter* abundance was lower in the subjects with progression of gastric lesions compared with non-progression group. Specifically, the remarkable decline in *Helicobacter* was observed after the progression to stage of dysplasia/GC compared with non-progression controls (Kadeerhan et al., 2021). The key non-*H. pylori* microbes associated with GC are summarized in **Table 1**. However, inconsistent results were found, necessitating additional validations.

**Table 1:** Key non-*H. pylori* microbes associated with gastric cancer.

PHYLUM	CLASS	ORDER	FAMILY	GENUS	SPECIES
*Firmicutes*	*Bacilli*	*Lactobacillales*	*Streptococcacaeae*	*Lactococcus*: potential harmful microbes for gastric mucosa (Coker et al., 2017; Ferreira et al., 2018; Hsieh et al., 2018)	*Lactococcus lactis*: potential beneficial microbes for gastric mucosa (Chen et al., 2019)
				*Streptococcus*: potential harmful microbes for gastric mucosa (Chen et al., 2019; Liu et al., 2019); also reported as potential beneficial microbes for gastric mucosa (Ferreira et al., 2018)	*Streptococcus anginosus*: potential harmful microbes for gastric mucosa (Coker et al., 2017; Liu et al., 2019) *Streptococcus infantis*: potential harmful microbes for gastric mucosa (Coker et al., 2017)
		*Bacillales*	*Bacillaceae*	*Bacillus*: potential harmful microbes for gastric mucosa (Kadeerhan et al., 2021)	
		*Lactobacillales*	*Lactobacillaceae*	*Lactobacillus*: potential harmful microbes for gastric mucosa (Ferreira et al., 2018; Hsieh et al., 2018)	*Lactobacillus brevis*: potential beneficial microbes for gastric mucosa (Chen et al., 2019) *Lactobacillus salivarius*: potential harmful microbes for gastric mucosa (Coker et al., 2017) *Lactobacillus fermentum*: potential harmful microbes for gastric mucosa (Coker et al., 2017)
	*Clostridia*	*Clostridiales*	*Clostridiaceae*	*Clostridium*: potential harmful microbes for gastric mucosa (Ferreira et al., 2018; Hsieh et al., 2018)	
*Bacteroidetes*	*Bacteroidetes*	*Bacteroidales*	*Prevotellaceae*	*Prevotella*: potential harmful microbes for gastric mucosa (Chen et al., 2019; Sung et al., 2020a; Kadeerhan et al., 2021); also reported as potential beneficial microbes for gastric mucosa (Ferreira et al., 2018; Gantuya et al., 2020)	*Prevotella melaninogenica*: potential harmful microbes for gastric mucosa (Liu et al., 2019) *Prevotella oris*: potential harmful microbes for gastric mucosa (Coker et al., 2017) *Prevotella intermedia*: potential harmful microbes for gastric mucosa (Coker et al., 2017)
*Proteobacteria*	*Betaproteobacteria*	*Neisseriales*	*Neisseriaceae*	*Neisseria*: potential beneficial microbes for gastric mucosa (Ferreira et al., 2018)	
*Fusobacteria*	*Fusobacteria*	*Fusobacterales*	*Fusobacteriaceae*	*Fusobacterium:* potential harmful microbes for gastric mucosa (Coker et al., 2017; Hsieh et al., 2018; Chen et al., 2019)	*Fusobacterium nucleatum*: potential harmful microbes for gastric mucosa (Coker et al., 2017)

Furthermore, based on the current findings, a panel of differential gastric bacteria can be developed to distinguish GC and the progression of GC with outstanding performance. A recently published meta-analysis, which enrolled six independent studies, reported that eight bacterial taxa could serve as a panel of biomarkers to discriminate GC from superficial gastritis with an area under the curve (AUC) of 0.850 (Liu et al., 2022). Regarding the progression of GC, Kadeerhan et al. reported a combination of four genera (*Bacillus*, *Capnocytophaga*, *Helicobater*, *Prevotella*) with age and sex to distinguish subjects after lesion progression from non-progression controls (AUC = 0.927) (Kadeerhan et al., 2021). In addition to a panel of bacteria, a new single index called Microbial Dysbiosis Index (MDI) has been presented. MDI is calculated by log (total abundance of genera increased in GC/total abundance of genera decreased in GC); a higher value of MDI means a higher risk of GC. The application of MDI has been applied in the evaluation of GC: the GC gastric microbiota had a higher MDI and the findings were confirmed in the validation cohorts (Ferreira et al., 2018).

## The different roles of *H. pylori* and non-*H. pylori* microbes in gastric carcinogenesis

**Figure 1:**
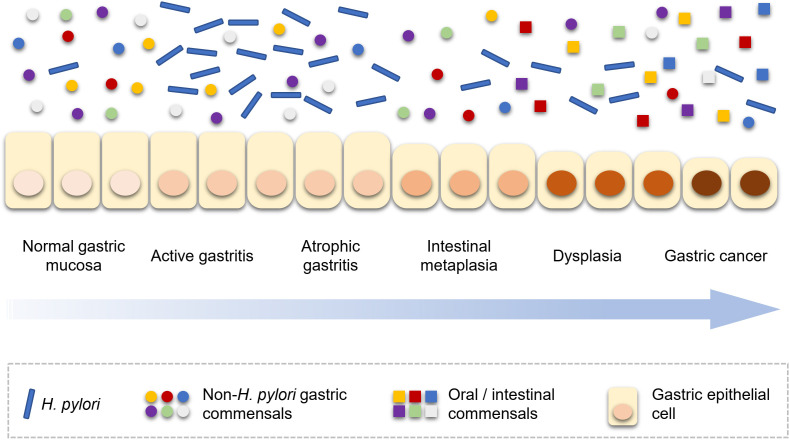
*H. pylori* and non-*H. pylori* microbes in the development of gastric carcinogenesis. *H. pylori*, *Helicobacter pylori*.

The progression of gastric carcinogenesis is detailed in **Figure 1**. Like bacterial driver-passenger model of colorectal cancer, the development of GC showed similar change pattern of gastric microbiota. Thus, *H. pylori* and non-*H. pylori* microbes show different roles in different stages of gastric carcinogenesis. First of all, the load of *H. pylori* in the stomach increases after the initial infection, especially in the active gastritis stage (Stewart et al., 2020). Interestingly, the *H. pylori* load decreases with the progression of gastric lesions. A population-based study showed that a lower *Helicobacter* abundance was observed in subjects with the progression of gastric lesions (Kadeerhan et al., 2021); another study reported that the abundance of *Helicobacter* was substantially lower in GC patients than gastritis (Ferreira et al., 2018). This phenomenon could be explained that, following *H. pylori* infection, due to the persistence of inflammation and the loss of acid-secreting parietal cells, the gastric environment becomes more favorable for the colonization of other bacteria and progression of lesions are accelerated (Polk and Peek, 2010). In detail, with the development of gastric lesions, oral or intestinal commensal microbes are enriched (Coker et al., 2017; Ferreira et al., 2018; Stewart et al., 2020). However, by the late stage of gastric precancerous lesions, the stomach environment is no longer suitable for *H. pylori* and the abundance *H. pylori* of decreases. This phenomenon has been confirmed in human studies (Ferreira et al., 2018; Kadeerhan et al., 2021). The key roles of *H. pylori* in different stages of gastric carcinogenesis were shown in the **Table 2**. In addition to the overall description of the progression of gastric carcinogenesis, the roles of certain bacteria remain to be clarified and further mechanism investigation is needed for a deeper understanding of this issue.

**Table 2:** Key roles of *H. pylori* in gastric carcinogenesis.

Stages in the development of GC	Descriptions
Uninfected stage	The major microbes in the healthy human stomach environment are *Firmicutes*, *Bacteroidetes*, *Actinobacteria*, *Fusobacteria*, and *Proteobacteria* (Guo et al., 2020; Guo et al., 2021) The abundance of *H. pylori* in the gastric microbiota of uninfected status is low (Guo et al., 2020; Guo et al., 2021).
*H. pylori*-dependent stage	*H. pylori*, as the “driver”, causes pathological changes of gastric mucosa and dysbiosis of gastric microbiota. After *H. pylori* infection, the stomach is dominated by *H. pylori* and accordingly, the abundances of non-*H.pylori* gastric commensals reduce substantially (Brawner et al., 2017; Das et al., 2017). *H. pylori* associated gastric microbiota dysbiosis includes: decreased microbial diversity, alterations in the microbial community structure, negative interactions between *H. pylori* and other microbes, etc. (Das et al., 2017; Llorca et al., 2017; Gantuya et al., 2019).
*H. pylori*-independent stage	With the progression of gastric lesions, the number of *H. pylori* decreases and the number of non-*H. pylori* microbes, as the “passengers”, increases correspondingly. The “passengers” are considered oral or intestinal commensal microbes (Coker et al., 2017; Ferreira et al., 2018; Stewart et al., 2020).

## Future perspectives

Non-*H. pylori* microbes and their interactions may also play a critical role in the development of GC. However, inconsistent findings were reported for non-*H. pylori* microbes associated with GC. Accordingly, further mechanism investigation is needed to validate these potential GC-associated non-*H. pylori* microbes, such as animal studies. Additionally, most human studies are case-control studies, which compared gastric microbiota of gastric mucosa between GC patients and control population. Due to this study design, we cannot infer a causal relationship between gastric microbiota dysbiosis and development and progression of GC. In other words, it is unclear whether gastric microbiota dysbiosis causes GC or whether GC causes gastric microbiota dysbiosis. Therefore, cohort studies with long-term follow-up are needed to confirm the major findings.

## Author contributions

YG drafted the manuscript, conceptualized the idea, and revised the manuscript. X-SC and M-GZ performed the literature search and revised the manuscript. YG and M-GZ contributed to drawing the figure. BY critically revised the manuscript and supervised the study. All authors contributed to the article and approved the submitted version.
